# Combining transcatheter arterial embolization with iodized oil containing Apatinib inhibits HCC growth and metastasis

**DOI:** 10.1038/s41598-020-59746-1

**Published:** 2020-02-19

**Authors:** Chen Zhou, Qi Yao, Hongsen Zhang, Xiaopeng Guo, Jiacheng Liu, Qin Shi, Songjiang Huang, Bin Xiong

**Affiliations:** 1 Department of Radiology, Union Hospital, Tongji Medical College, Huazhong University of Science and Technology, Wuhan, 430022 China; 2 Hubei Province Key Laboratory of Molecular Imaging, Wuhan, 430022 China; 3 Henan Cancer Hospital Zhengzhou, Henan, China

**Keywords:** Cancer metabolism, Targeted therapies, Tumour angiogenesis

## Abstract

Transcatheter arterial embolization (TAE) plays an important role in clinical liver tumor therapy. However, hypoxia after TAE limit the medium-long term efficacy of TAE. Thus, in our study, we explored the treatment effect and mechanism of combining transcatheter arterial embolization with adopted iodized oil containing Apatinib on suppressing tumor growth and metastasis. We simulated the changing of tumor microenvironment before and after TAE both *in vitro* and *in vivo* models. The anti-angiogenic effect of Apatinib was explored by bioassays in human umbilical vein endothelial cells (HUVECs), including cell migration, invasion and apoptosis, tube formation, and wound healing. Further experiments showed that Apatinib inhibited tumor microangiogenesis to achieve the aims of inhibiting tumor growth and recurrence by means of down-regulating the phosphorylation of the RAF-mek-erk, PI3K-akt and P38MAPK pathways. The antitumor growth and anti-angiogenic effect of Apatinib was further validated by the animal experiment. Taken together, we concluded that Apatinib inhibits the angiogenesis and growth of liver cancer by down-regulating the PI3K-akt, RAF-mek-erk and P38MAPK pathways, and has a stronger inhibitory effect in hypoxic environments. Combining TAE with adopted iodized oil containing Apatinib has a stronger inhibitory effect in VX2 liver tumor growth and metastasis, which suggesting such combinations may provide a new target and strategy for interventional therapy of liver cancer.

## Introduction

Hepatocellular carcinoma (HCC) is garnering more research and clinical attention as the third-leading cause among cancer patients^1,2^. Due to the concealment of the onset of liver cancer, most patients are already in the advanced stage at the time of diagnosis. Transcatheter arterial embolization (TAE) is playing a major role in the therapy for patients who are not candidates for surgery^3,4^. However, although TAE therapy prolongs the survival time of many patients^5^, the inability of conventional TAE therapy with iodine oil to effect thorough necrosis of the tumor cells should not be ignored^6,7^. Incomplete necrosis of the tumor could aggravate hypoxia in the tumor^8,9^. And hypoxia can further activate angiogenesis and tumor growth, which often associated with the tumor metastasis and recurrence, and is the critical factor limiting the treatment effect of TAE^10,11^. Hence, it is mightily vital to explore new strategies for HCC treatment.

Malignant angiogenesis is believed to be the most crucial aberrantly activated pathway; this belief is highlighted by the nature of HCC as a hypervascular tumor^12,13^. Tumors often remain hypoxic due to decreased blood flow, leading to the sustained overproduction of vascular endothelial growth factor (VEGF) after TAE therapy^14,15^. VEGF is a critical factor that induces developmental angiogenesis via VEGF receptor (VEGFR)-dependent signaling, which in turn leads to tumor recurrence and metastasis^16,17^. VEGFR2 is mainly expressed on endothelial cells, mediating the angiogenic effects of VEGF^18,19^. However, this neovascularization produces abnormal leaky vessels that produce interstitial hypertension, edema and tumor hypoxia. This process forms a vicious circle of nonproductive angiogenesis, tumor growth and hypoxia after TAE^20^. Accordingly, suppression of the VEGF signaling pathway employing VEGFR2 tyrosine kinase inhibitors has become a hopeful therapeutic strategy to decrease excessive angiogenesis in HCC after TACE.

Apatinib (YN968D1), a novel tyrosine kinase inhibitor, is a highly selective VEGFR-2 inhibitor, with a binding affinity 45 times that of sorafenib^21,22^. Apatinib has antineoplastic and antiangiogenic activities in gastric cancers, colon, breast, non-small cell lung and so on^22–24^.

Herein, we hypothesize combining iodized oil containing Apatinib with TAE has its potential activities in treating HCC. Owing to our limited understanding of the molecular mechanisms of Apatinib for HCC treatment and the Apatinib-mediated downstream pathways in HCC cells, further detailed studies are needed to elucidate the mechanism of Apatinib in HCC. Together, these results will be of great benefit to the strategies against human HCC.

## Methods and Materials

### *In vitro*

#### Materials

HepG2 was maintained in Dulbecco’s modified Eagle’s medium (DMEM, Gibco, US) with 10% fetal bovine serum (FBS), 100 mg/mL penicillin, and were cultured at 37 °C with 21% O_2_ (normoxic conditions), and hypoxic conditions were simulated 24 h by incubation in RPMI 1640 medium containing 200 μM CoCl_2_^25^. Primary antibody was obtained from Bioss (China); second antibody was obtained from Aspen.

#### Study design

Human umbilical vein endothelial cells (HUVECs, ATCC, Manassas, VA) were cocultured with HepG2 cells under normoxic or hypoxic conditions on Matrigel. The study design included 8 treatment groups: Groups A, B, C, and D were normoxic matrices with different concentrations of Apatinib (Hengrui Medicine Co., Jiangsu) (0, 1, 10, and 50 μmol/L), and groups E, F, G, and H were hypoxic matrices with different dose of Apatinib (0, 1, 10, and 50 μmol/L). All experimental methods were ratified by the Animal Experiment Committee of Institute for Huazhong University of Science and Technology. And we confirmed that all experiments were performed in accordance with relevant guidelines and regulations.

#### Viability (CCK-8 assay)

HepG2 cells were cultured and plated into 96-well flat bottom plates (100 mL per well) for 24 h under normoxic or hypoxic conditions. The cells were then supplemented with Apatinib at concentrations of 0, 0.1, 1, 10 or 50 µM. After 48 hours of treatment, CCK-8 solution(Beyotime Biotechnology, China) was added and incubated at 37 °C for 4 hours. Cells were cultured for 1–4 h under the above conditions, and measured using microplate reader (Diatek, DR-200Bs, CA, USA) with absorbance at 450 nm.

#### Migration and invasion assays

First draw a parallel line on the back of the 6-well plate with a marker pen and traverse the hole. 2 mL of medium containing approximately 5 × 10^5^ cells was added to each well. After the cells are covered with the bottom of the well, the cells are crossed with the gun head perpendicular to the horizontal line of the marker pen. The smeared cells were gently rinsed with PBS and added to the medium. The cells were cultured in a cell culture incubator, and the well plates were taken out at 0 and 24 hours for photographing under an inverted microscope (Olympus).

200 ul of serum-free cell suspension containing 10^5^ cells was taken into the transwell chambers (Corning, USA). Subsequently, 500 µL of complete medium containing 10% FBS was added to the 24-well plate, and the chamber was placed in the plate. Incubate for 48 h at 37 °C in a CO_2_ (content 5%) incubator. The stain was prepared and stained and photographed on the non-cell inoculated side.

#### Matrigel-based tube formation assay

Via a precooled tip, 50 µL of liquid Matrigel (Corning, 354248, USA) was embedded into a 96-well plate at 4 °C. A total of 2 × 10^4^ HUVECs in 100 µL of complete medium containing different concentrations of apatinib were plated on the solidified Matrigel suspension. Twelve hours after the HUVECs were overlaid, the cocultures incubated on Matrigel (37 °C, 5% CO2) were inspected and photographed at 200 × magnification. Check 5 separate fields per well and calculate average tubes/field.

#### HUVEC flow cytometry apoptosis assay

PBS was prechilled at 4 °C and diluted with an appropriate amount of binding buffer. Then, the cells were collected in a flow tube and rinsed twice with PBS. Cell pellet resuspended in 300 µl Binding Buffer. Subsequently, 5 µL of annexin V-FITC was added, mix and incubate for 10 min in the dark. 5 µL PI was added, mixed and incubated. On-board detection within 1 h, FITC excitation 494 nm emission 520 nm, PI excitation 493 nm emission 636 nm.

#### WB analysis

Cells were resuspended, centrifuged, and the cell pellet was collected. Add Protease Inhibitor Cocktail(ROCHE) and cell protein extraction reagent, repeatedly beat upon with a pipette to ensure complete cell lysis. After centrifugation at 13,000 g for 5 min at 4 °C, the supernatant was collected, which was the total protein solution. The sample protein concentration was determined using a BCA protein concentration assay kit (Aspen, Guangzhou, China), and ensure that the total amount of protein in each sample is 40 ug. Separation of proteins by SDS-PAGE (Aspen, Guangzhou, China) electrophoresis. And transferred onto PVDF membranes (Millipore, Darmstadt, Germany). Add primary and secondary antibodies for antibody incubation. The freshly prepared ECL (Aspen, Guangzhou, China) mixed solution was added dropwise to the protein side of the membrane, and exposed in a dark room. The film was scanned and archived, and the optical density of the target bands was analyzed by the AlphaEaseFC software processing system.

#### Immunofluorescence

HUVECs were added on sterilized coverslips in 6-well plates and treated according to the groups. 4% paraformaldehyde was fixed for 30 min and permeabilized using 3% H2O2 for 20 min. The cells were covered with a 5% BSA diluted primary antibody and incubated in a humidifying box overnight. Following incubation with a FITC- or Cy3-labeled goat anti-rabbit secondary antibody for 50 min, 50–100 ul of DAPI stain was added dropwise to each well and incubated for 5 min. Add anti-fluorescence quencher to the cells, cover the slides, and observe under a fluorescence microscope.

### *In vivo*

#### Study design and animal model

The 2.5–3.0 kg adult New Zealand white rabbits used in this study were all purchased from the Animal Experimental Center of Huazhong University of Science and Technology. All experimental plans were ratified by the Animal Experiment Committee of Institute for Huazhong University of Science and Technology.

The study design included 4 treatment groups and ten rabbits per group: the sham (NS) group (treatment with 5 mL of saline without Apatinib or ultra liquid iodized oil); the AI group (treatment with both 3 mL of lipiodol containing 50 mg of Apatinib and TAE); the I group (treatment with both 3 mL of lipiodol supplemented with a gelatin sponge); and the A group (treatment with a mixture of 3 mL of saline with 50 mg of Apatinib suspension).

The rabbit’s abdominal cavity was cut about 4 cm to reveal the left medial liver lobe. A piece of 1 mm^3^ VX2 tumor tissue, take from tumor-bearing rabbits, was implanted into left medial lobe of the liver approximately 0.5 cm in depth, and then covered by gelatin. At 16 days after implantation, the tumor sizes were measured by CT scan and the rabbits carrying tumors of 10- 20 mm in diameter were used for subsequent experiments.

#### TAE procedure

All rabbits were anesthetized, and femoral artery was dissected and catheterized. Then, catheter was super-selectively inserted to the HCC feeding artery from the femoral artery under the digital subtraction angiography (DSA). Subsequently, the drugs were injected into the catheter according to the different groups. Finally, the femoral artery was sutured, and all rabbits were intramuscularly injected with penicillin daily for three days.

#### Tissue sample harvesting

Tumor growth was monitored with contrast-enhanced CT on days 0 and 7. After scanning, all the images acquired were processed by the Syngo Fastview image processing system, and the size, location, shape and the presence of necrosis and intrahepatic metastasis of the implanted tumor were analyzed by twos senior doctors of radiology department. The maximum diameter (D_1_) and transverse diameter (D_2_) of tumor were separately recorded and Tumor volume was calculated by using the following modified formula for elliptic volume: V = D_1_ × D_2_^2^/2. The tumor growth rate was calculated by using the following formula: V_7_/V_0_ * 100%

All animals were euthanized at 1 day, 3 days or 7 days after TAE. Tumor and surrounding liver tissues were removed, measured, formalin-fixed, paraffin embedded, incised continuously. Immunohistochemical analysis was performed and slides were stained with hematoxylin-eosin.

### Statistical analysis

All data are presented as the means ± SDs. Measurement data were analyzed statistically using one-way ANOVA and double-factor variance analysis. The results were considered statistically significant at a P-value of <0.05. All figures were generated in GraphPad prism V7.0.

## Results

### Combining TAE with adopted iodized oil containing Apatinib has a stronger inhibitory effect in VX2 HCC model

**Figure 1:**
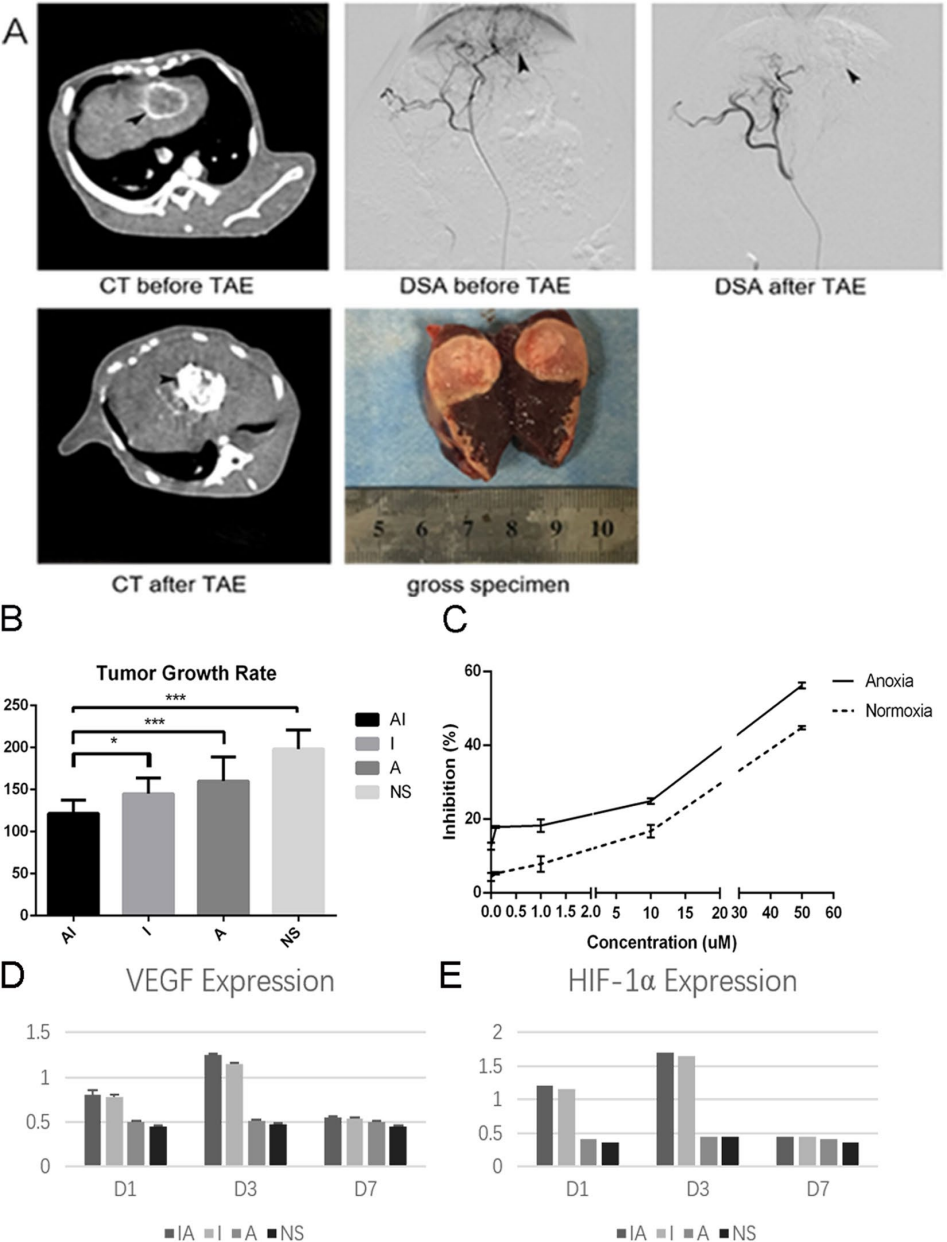
TAE combined with iodized oil containing Apatinib can inhibit tumor growth in rabbit VX2 liver cancer model. (**A**) CT enhancement scan and DSA angiography aimed at liver cancer were performed on rabbits before and after intervention embolization treatment. We could see round tumor in the liver (black arrows), and that the blood-supply vessels in tumor were completely blocked after treatment. Gross specimens of the liver were taken 7 days after embolization, tumor was almost completely necrotic after embolization. (**B**) TAE combined with Apatinib can inhibit the growth of liver cancer. P = 0.022 < 0.05 versus NS group, P = 0.000 < 0.001 versus I group. (**C**) Apatinib inhibite HepG2 cell proliferation Data from the CCK-8 assays show the inhibition of HepG2 cell proliferation in response to treatment with different concentrations of Apatinib under normoxic and hypoxic conditions for 48 h. (**D,E**) The expression of VEGF and HIF-1α varied with time in each group of experimental rabbits. Each point represents the mean ± SD (n = 3). *P < 0.05, ***P < 0.001.

To verify the effect of combining TAE with iodized oil containing Apatinib on reducing HCC growth ability, interventional embolization assay was performed in VX2 tumor-bearing rabbits. *In vivo*, experimental data shows that the group AI GRs was obviously below the other groups (P = 0.012 < 0.05) (Fig. 1A,B). The data revealed that compared with lipiodol embolization alone, TAE therapy combined with Apatinib treatment yielded a greater inhibitory effect on tumor growth in the VX2 liver cancer model. Consistent with the results of the vivo result, Apatinib showed dose-dependent inhibition of HepG2 cell proliferation after treatment with Apatinib at concentrations ranging from 0 to 50 µM, and that Apatinib induces superior tumor growth inhibition in anoxic environments than in normoxic environments (Fig. 1C). The expression of VEGF and HIF-1α was significantly increased after TAE in the VX2 HCC model groups compared with that in the placebo group (P < 0.05), especially after 3 days (P < 0.001). And in the AI group and the I group, the expression of HIF-1α and VEGF was significantly higher than that in the other two groups on day 3, and there are no significant difference between-group AI and I (Fig. 1D,E). It confirmed that the overproduction of VEGF and HIF-1α after embolization.

### Apatinib inhibited angiogenesis, especially in a hypoxic environment

**Figure 2:**
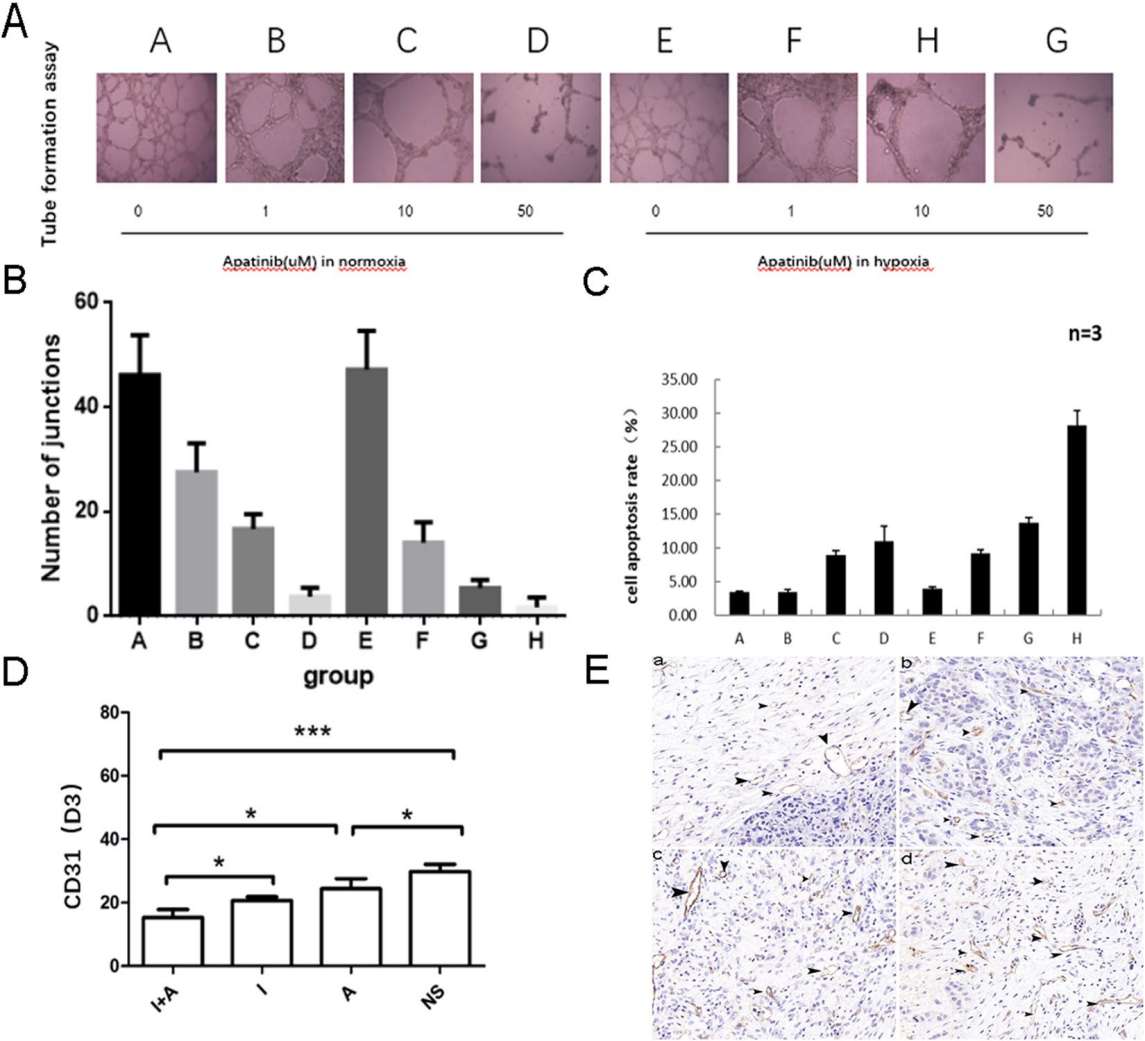
Apatinib significantly suppressed angiogenesis of tumor cells in a dose-dependent manner (A–D). (**A**) The tube formation of HUVECs with different concentrations of Apatinib under normoxic or hypoxic conditions. (**B**) The number of junctions of each group. Apatinib could inhibit the HUVECs proliferation at a very low dose (0.1 uM), especially in the hypoxic environment, P > 0.05 in group A vs E and P < 0.05 in group B vs F, C vs G, D vs H. (**C**) The cell apoptosis rate of HUVECs with with different concentrations of Apatinib under normoxic or hypoxic conditions, P > 0.05 in group A vs E and P < 0.05 in group B vs F, C vs G, D vs H. (**D**) CD31 in VX2 tumor tissues was markedly inhibited by Apatinib in Day 3 (D3), the CD31 of group AI was lower than that of group I (P = 0.027 < 0.05). (**E**) Images of MVD in each group was shown by light microscopy (CD31 staining, 200×), a-Group AI, b-Group I, c-Group A and d-Group NS. Stripe and annular microvessels (black arrows) were observed in all the four groups, the MVD in Group AI was significantly lower than the other groups. Each point represents the mean ± SD (n = 3). *P < 0.05, ***P < 0.001.

We can see from the figure that a concentration-dependent manner in which Apatinib inhibits basal tube formation and HUVEC proliferation agents from 0 to 50 μM, especially in the hypoxic environment (P = 0.000 < 0.001) (Fig. 2A,B). The apoptosis assay results also indicated that HUVEC apoptosis was activated by Apatinib in a dose-dependent manner. Furthermore, apoptosis was more highly induced in hypoxic conditions(P = 0.000 < 0.001) (Fig. 2C). These data suggested that Apatinib inhibits angiogenesis.

In addition, *in vivo* experiments also proved this. Immunohistochemical staining for CD31 expression, which is known to be highly correlated with tumor angiogenesis, was carried out to investigate the expression of tumor MVD. Consistent with the vitro model, the CD31 staining data revealed that the AI group had a significantly lower tumor MVD than the other three groups (P < 0.01) (Fig. 2D,E), which suggested that combining TAE with iodized oil containing Apatinib could inhibit the process of tumor angiogenesis.

### Apatinib suppressed the metatasis of HUVECs in a concentration-dependent manner, especially in hypoxic environments

**Figure 3:**
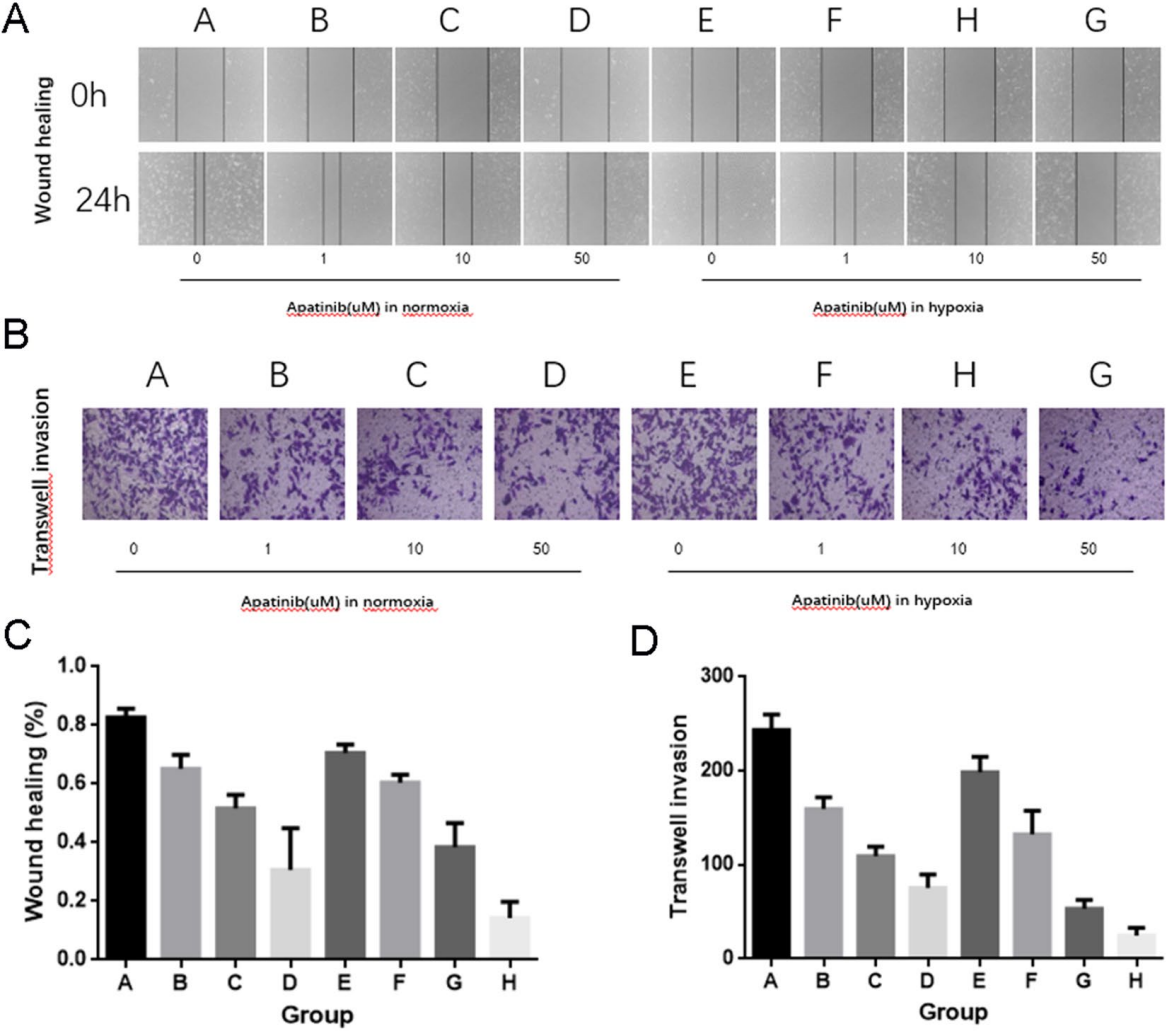
Apatinib inhibited the migration and invasion of HUVECs. (**A,C**) Cell migration was assessed by a wound healing assay, P > 0.05 in group A vs E and P < 0.05 in group B vs F, C vs G, D vs H. **(B,D)** Consistent with the result of wound-healing assay, the invasion property of HUVECs was greatly suppressed by Apatinib, which measured by Matrigel-coated Transwell assay, P > 0.05 in group A vs E and P < 0.05 in group B vs F, C vs G, D vs H. The data are expressed as the means S.D.

Wound-healing and migration assays were performed for evaluating the control effect of Apatinib on migration and invasiveness of HUVECs. Apatinib showed inhibition of HUVECs at a very low dose (1 µM). Subsequently, after HUVECs treated with 10 and 50 μM Apatinib for 24 h, the invasion reduced by 43% and 56%, respectively, under normoxic conditions, and by 49% and 79%, respectively, under hypoxic conditions (Fig. 3A,C). What confirm the results of the wound healing assay is that HUVEC migration across the Transwell membrane was greatly suppressed by Apatinib in a concentration-dependent manner (1, 10, and 50 μM), especially under hypoxic conditions (Fig. 3B,D). The data suggested that Apatinib is an inhibitor of migration and invasion in HUVECs, especially under hypoxic conditions.

### Apatinib regulated PI3K-Akt, RAF-MEK-ERK and P38 MAPK signaling pathway-related molecules

**Figure 4:**
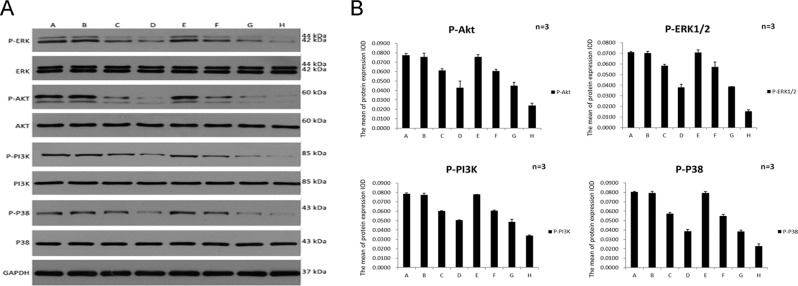
(**A**) Effects of Apatinib on the phosphorylation of various growth factor-stimulated receptors at the cellular level detected by Western blot analysis. Phosphorylation of Akt, ERK, PI3K and P38 was suppressed by Apatinib in a dose-dependent manner, and the suppression was more significant in the hypoxic environment, the Western Blot original data are shown in the supplementary information. (**B**) immunofluorescence data of HUVEC treated with Apatinib, P > 0.05 in group A vs E and P < 0.05 in group B vs F, C vs G, D vs H. Each point represents the mean ± SD (n = 3).

The pathway downstream of VEGF-VEGFR, such as PI3K-Akt, RAF-MEK-ERK and P38 MAPK, also mediate metatasis and proliferation of endothelial cell. To confirm whether the pathway was involved in the antiangiogenic responsiveness of Apatinib, the phosphorylation of some kinases were inspected by Western blot analysis. The experimental results revealed that the phosphorylation levels of ERK, Akt, PI3K and P38 were decreased in HUVECs after treated with Apatinib (Fig. 4). Apatinib significantly reduced the VEGF-induced phosphorylation of Akt, ERK, PI3K and P38 in HUVECs in a dose-dependent manner, especially under hypoxic conditions. Interestingly, the four kinases demonstrated similar sensitivity to Apatinib-induced inhibition.

The blockade of these kinases by Apatinib was so drastic that we further verified the reliability of the results by immunofluorescence. The immunofluorescence results indicated that Apatinib potentially exerted its inhibitory effects on HUVECs by decreasing activity in the PI3K-Akt, RAF-MEK-ERK and P38 MAPK signaling pathways (Fig. 4).

## Discussion

TAE, as mentioned above, has been widely used to treat advanced HCC, and there was no difference between the TAE with or without doxorubicin^26^. But because of recurrence and metastasis due to the highly hypoxic microenvironment after TAE, the outcomes remain modest. It is necessary to further study the effect of interventional embolization on the angiogenesis of HCC and the corresponding intervention for the changes of angiogenesis after embolization. Up to now, there is no report on the use of adopted iodized oil containing VEGFR-2 inhibitor Apatinib in interventional therapy of hepatocellular carcinoma. Therefore, we propose a new concept of interventional embolization combined with adopted iodized oil containing Apatinib targeted therapy of liver cancer. The results show that this new administration protocol successfully interrupted cell migration and the recurrence of VX2 liver tumors.

Angiogenesis, a hallmark of tumor development, is essential for cancer invasion and migration^15^. Realizing the limitations of TAE due to hypoxia, more and more studies have explored new ways of vascular normalization to treat HCC. Thus, the antiangiogenic impact of Apatinib was further validated in HUVECs by bioassays, including cell migration, invasion and apoptosis, tube formation, and wound healing assays. These results revealed that Apatinib inhibits HUVEC migration and invasion, especially in hypoxic environments. The results of vivo experiments also confirmed that there were significant differences between the Group A and Group NS in tumor growth rate and MVD. Comprehensively, our results indicate that Apatinib has great potential as a pharmaceutical treatment for human HCC by inhibiting cell migration, invasion and survival.

Angiogenesis is critical for the proliferation and metastasis of HCC, and a high level of MVD, which is demonstrated a harmful prognosis marker for HCC patients and related to a reduction in patient survival^27,28^. Moreover, the interaction between VEGF and VEGFR2 supports HCC cell growth and migration through an angiogenesis-independent antiapoptotic pathway^29–31^. However, the drugs that block VEGFA, which is considered a major VEGF involved in angiogenesis, have not achieved tiptop results^4^; therefore, Apatinib, a potent VEGFR-2 inhibitor, is considered. In this study, we had simulated the changing of tumor microenvironment before and after TAE both *in vitro* and vivo models. We found out that overproduction of VEGF and HIF-1α after embolization, and there are no significant difference between-group AI and I. But we demonstrated that Apatinib mixed with lipiodol to enter the tumor through the hepatic artery significantly reduced the MVD. In other words, Apatinib plays an antiangiogenic role after interventional embolization, blocking the regeneration of blood vessels. And this new administration protocol could significantly suppress the GR of VX2 liver tumors; this effect was further confirmed by the inhibition of HepG2 cell proliferation by Apatinib in the CCK-8 assays. Interestingly, we found that Apatinib was more potent against HepG2 cell proliferation in a hypoxic environment than in a normoxic environment, in a dose-dependent manner. Thus, we concluded that Apatinib has a direct toxic effect on HCC cells, that Apatinib can effectively inhibit tumor cell proliferation in a concentration-dependent manner, especially in the anoxic environment. And this is the reason why Apatinb can work efficient with TAE.

What is the major gap in the knowledge about TAE is that the incomplete understanding of the mechanism between antiangiogenesis and tumor growth. Insight into this mechanism represents a necessary foundation on which to increase the effectiveness of TAE treatment paradigms. HIF-1α is elevated after hypoxia and triggers the VEGF/VEGFR pathway, thereby induceing genes expression involved in cell proliferation and angiogenesis. Therefore, exploring the effect of Apatinib on these downstream pathways is very important. The PI3K/Akt signaling pathway, overactivated in many malignant tumor, is playing a vital role in various cellular processes involved in angiogenesis in endothelial cells. In addition to the PI3K/Akt signaling pathway, the VEGF/VEGFR pathway activate endothelial cell proliferation and metastasis by inducing other downstream kinases, such as P38, ERK and FAK. Indeed, the Western blotting results demonstrated that Apatinib downregulated the protein levels of p-ERK, p-Akt, p-PI3K and p-P38 in a concentration-dependent manner, especially in a hypoxic environment, which suggesting that the mechanism underlying the antiangiogenic effect of Apatinib is blocking the activation of these kinases.

In summary, Apatinib can effectively block the VEGF-VEGFR pathway by downregulating the activity of the RAF-MEK-ERK, PI3K/Akt, and P38 MAPK signaling pathways, which contributed to inhibiting the growth, invasion and migration of the residual tumor after embolization in an anoxic microenvironment. And these findings made it a promising potential therapy of combining TAE therapy with the VEGFR-2 inhibitor Apatinib for the treatment of HCC. And our efforts have deepened the study of interventional treatment of liver cancer into the field of the tumor microenvironment and hopefully provide a new target and strategy for interventional therapy of liver cancer.

## Supplementary information

Supplementary information.
