# Supplementary material for: Combining transcatheter arterial embolization with iodized oil containing Apatinib inhibits HCC growth and metastasis
Source: Sci Rep. 2020 Feb 19;10:2964. doi: 10.1038/s41598-020-59746-1 (PMC7031235; doi:10.1038/s41598-020-59746-1)
Supplement: Supplementary file 1 — Supplementary information. [file 41598_2020_59746_MOESM1_ESM.pdf]

Title

Combining transcatheter arterial embolization with iodized oil containing Apatinib inhibits HCC growth and metastasis

Western Blot original data

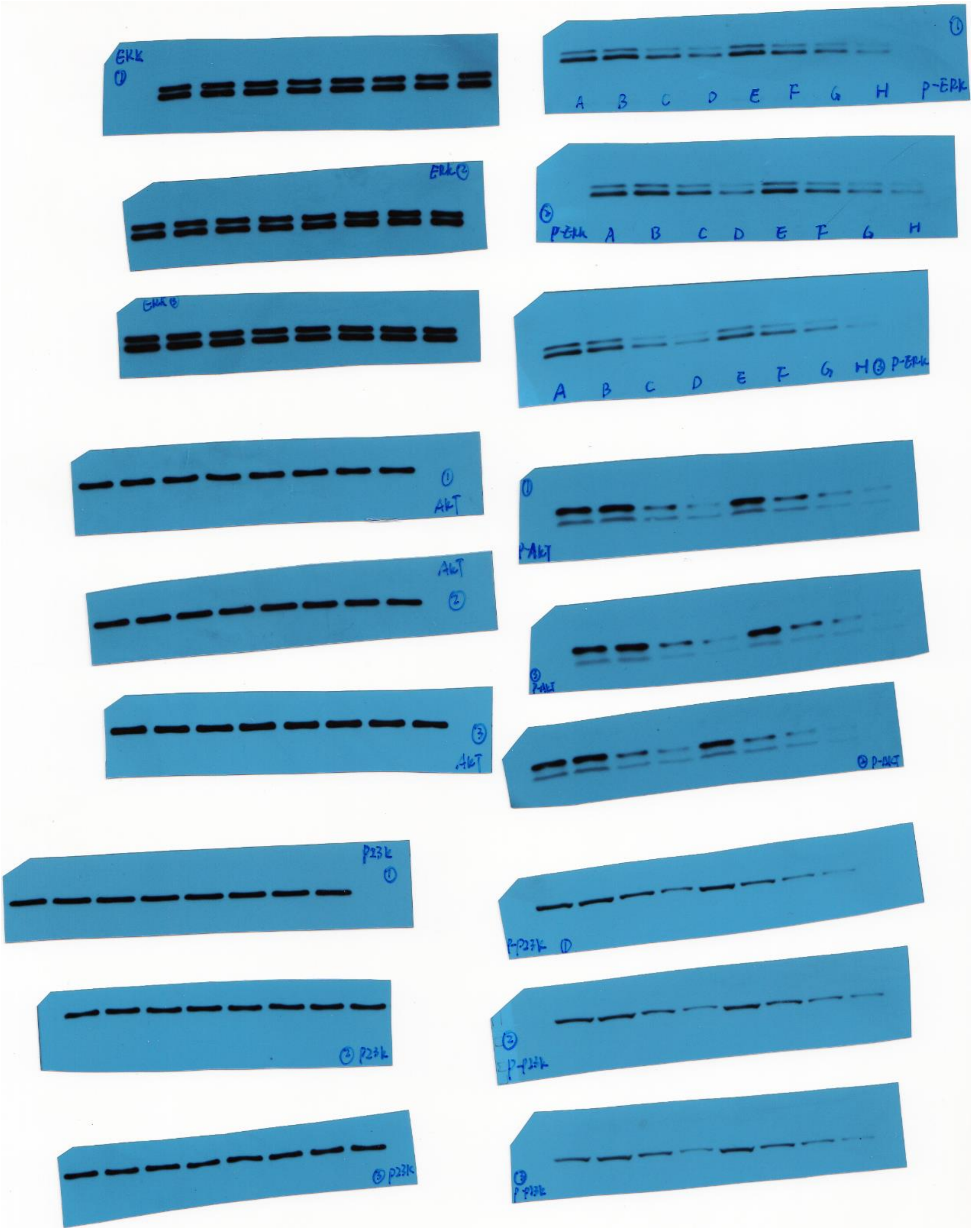

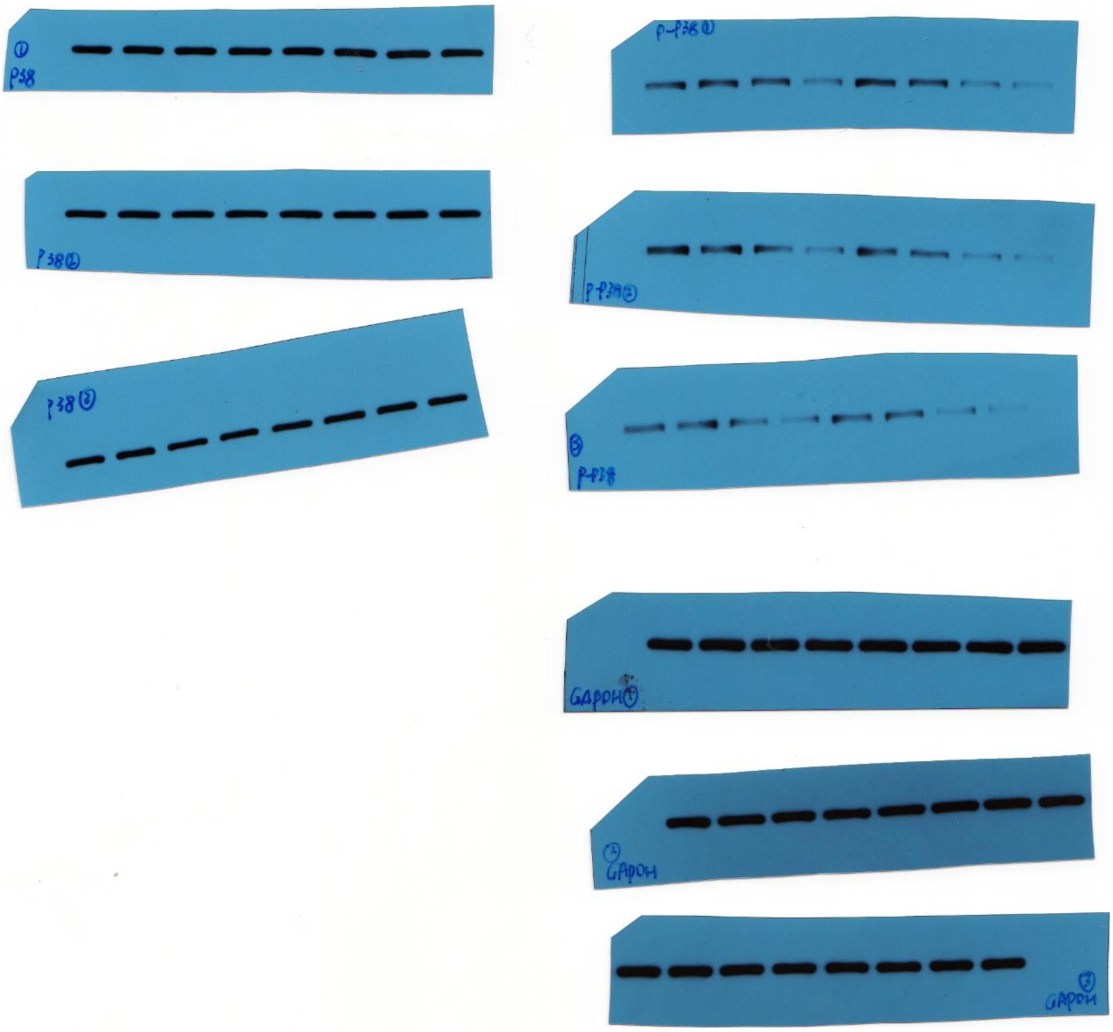
① P38
P38 ②
P38 ③
P-P38 ①
P-P38 ②
③ P-P38
GAPDH ①
② GAPDH
③ GAPDH
